# Hepcidin gene polymorphisms and iron overload in β-thalassemia major patients refractory to iron chelating therapy

**DOI:** 10.1186/s12881-020-01011-3

**Published:** 2020-04-08

**Authors:** Parinaz Zarghamian, Azita Azarkeivan, Ali Arabkhazaeli, Ahmad Mardani, Majid Shahabi

**Affiliations:** 1 Blood Transfusion Research Center, High Institute for Research and Education in Transfusion Medicine, Hemmat Expressway, IBTO Building, Tehran, 1449613111 Iran; 2 Department of Hemovigilance, Iranian Blood Transfusion Organization, Tehran, Iran

**Keywords:** β-Thalassemia, Iron overload, Hepcidin, SNP

## Abstract

**Background:**

β Thalassemia is one of the most common groups of hereditary haemoglobinopathies. Affected people with thalassemia major are dependent on regular blood transfusion which on the long term leads to iron overload. Hepcidin is a peptide hormone and an important regulator of iron homeostasis, especially in thalassemia. Expression of this hormone is influenced by polymorphisms within the hepcidin gene, HAMP. Several studies emphasized the role of single nucleotide polymorphisms (SNPs) located in the promoter region of the gene. This study aimed to analyze the association between three SNPs in promoter of HAMP, c.-582A > G, c.-443C > T, and c.-153C > T, with iron overload in β-thalassemia major patients.

**Methods:**

A total of 102 samples from β thalassemia major patients were collected. Genomic DNA was extracted and segments of DNA encompassing rs10421768 and rs142126068 were sequenced. Statistical analysis was performed by SPSS Statistics 23 using independent t test and Fisher’s exact test.

**Results:**

A total of 102 adult β-thalassemia major patients were genotyped for three SNPs in the promoter region of HAMP gene by PCR and direct sequencing. Most of the patients (71.3%) were iron overloaded (based on plasma ferritin > 1000 ng/ml) in spite of receiving regular iron-chelating therapy. Our analysis revealed a statistically significant difference between the level of cardiac iron accumulation and c.-582A > G variant (*p* = 0.02). For c.-443C > T statistical analysis was on the edge of the significant relationship between the minor allele and serum ferritin (*p* = 0.058). All samples were homozygous for allele C of c.-153C > T.

**Conclusions:**

Despite chelating therapy, iron overload is still one of the main complications of thalassemia. Our findings and others emphasize the role of hepcidin -582A > G polymorphism as a key component of iron homeostasis in these patients.

## Background

β-thalassemia is one of the most predominant form of thalassemia in which the production of hemoglobin β chain is impaired quantitatively [1]. β-thalassemia is characterized by anemia [2] and depending on the severity of the anemia, the disease is classified into three categories, namely major, intermedia, and minor [3]. The average frequency of thalassemia gene in Iran is higher than the global mean [4] because some area of the country including the Caspian Sea and the Persian Gulf and Oman Sea coasts are in the middle of the so-called thalassemia belt. These are the areas that were endemic for malaria in the past [5].

The gene encoding the β chain is on chromosome 11p15.4 and contains three exons and two introns [6]. At the molecular level, IVS-II-1 (G → A) is the predominant mutation found [7].

Affected people with thalassemia major are dependent on blood transfusion which has greatly improved the quality of life in these patients. The long-term transfusion would lead to iron overload and its consequences including heart failure, growth retardation, endocrine problems and splenomegaly [2, 8–10].

Iron metabolism is a complex process and is controlled by several mechanisms mainly targeting intestinal absorption [11]. Hepcidin, a key element in iron hemostasis, is a small antimicrobial peptide and encoded by the HAMP gene on 19q13, which contains 2637 bp and comprises three exons [12]. It is predominantly expressed in the liver and acts by reducing the expression of ferroportin (FPN), an iron transporter on the intestinal cell surface. FPN is mainly expressed in duodenal enterocytes, liver Kupffer cells, periportal hepatocytes, and splenic macrophages. Hepcidin-FPN complex is regarded as a central negative regulator of iron egress from hepatocytes [13]. Hepcidin influences iron homeostasis by three mechanisms; 1) inhibition of iron absorption from the duodenum, 2) blocking the release of recovered iron from macrophages and 3) controlling the movement of iron stores in hepatocytes [14]. These lead to a decrease in the iron outflow from its storage resulting in trapping of iron in the cell, maintaining the plasma iron level within normal range [15]. The level of hepcidin in thalassemic patients is low which is mainly due to decreased synthesis in the liver and is also affected by hematopoiesis, anemia and iron overload [16]. Erythroferrone (ERFE), an erythroid regulating peptide, inhibits hepcidin transcription and increases iron acquisition through intestinal absorption [17].

Several studies have revealed that the expression of hepcidin is influenced by single nucleotide polymorphisms located in the promoter region of HAMP [18–20]. Parajes et al found c.-582A > G variant decreases the expression of HAMP [20]. Andreani et al found that c.-582A > G variant is associated with liver iron overload and increased serum ferritin level in β-thalassemia major patients with irregular chelating therapy [18]. Island et al demonstrated that c.-153C > T variant, which is located in a BMP-responsive element, reduces basal hepcidin gene expression by impairing its response to BMPs and IL-6 [19].

This study aimed to analyze the association of c.-582A > G (rs10421768), c.-153C > T (rs142126068), and with iron overload in β-thalassemia major patients on regular iron-chelating therapy. The c.-443C > T variant (rs117345431) was later included in the study when sequencing data revealed heterozygosity in that locus.

Although several studies have been conducted on molecular genetics of thalassemia in Iran, to the best of our knowledge, this is the first molecular study of hepcin gene in β-thalassemia major in our country.

## Methods

### Patients

This cohort study analyzed one hundred and two patients affected by β-thalassemia major who referred to Zafar Thalassemia Clinic of Tehran, Iran. All participants signed informed consent. Inclusion criteria were considered as regular transfusion (2-4 week interval), regular iron chelating therapy, and age above 14 years. Iron overload status was defined as ferritin level more than 1000 ng/ml as explained by TIF^1^ [21].

Chelation therapy at the time of the sample collection was deferoxamine (Desferal®) in 36 cases, deferasirox (Exjade®) in 6 cases, the combination of deferoxamine (Desferal®)/deferiprone (Ferriprox®) in 50 cases, and deferoxamine (Desferal®)/deferasirox (Exjade®) in 10 cases.

### Iron status assessment

Heart and liver iron concentration were measured byT2*MRI (MRI 1.5 Tesla, Achieva, A-series, Philips, Netherland). Results of cardiac T2* were categorized as severe (T2* < 10 ms), moderate (10 < T2* < 14 ms), mild (14 < T2* < 20 ms), and normal (T2* > 20 ms) The results of liver T2* were defined as normal (T2* > 30 ms), mild (T2* > 6.2 ms), moderate (3.1 < T2* < 6.2 ms), severe (2.1 < T2* < 3.1 ms), and very severe (T2* < 2.1 ms) [22]. Serum ferritin was determined by a electrochemiluminescence immunoassay (Elecsys Ferritin, Roche Diagnostics International Ltd., Switzerland).

### Molecular analysis

#### DNA extraction

Three ml of EDTA-blood was lysed with 12 ml of lysing buffer (NH_4_CL 150 mM, NaHCO_3_ 10 mM, EDTA 1 mM) and centrifuged at 3000×g for 10 min. The supernatant was discarded and the WBC pellet was washed with normal saline twice. Genomic DNA was extracted from 0.2 ml of WBC with a commercial assay (GeneAll® Exgene™ Blood SV mini, Seoul; Korea) according to the manufacturer’s instruction.

An 803 bp fragment of DNA containing hepcidin promoter region was amplified with specific primers (Table 1). PCR products were cleaned and sequenced with Bigdye assay (Big dyes Terminator Version 1.1; Applied Biosystems, Weiterstadt, Germany). DNA sequences were aligned and analyzed using NCBI accession number NG_011563.2 as the reference sequence.

**Table 1:** Characteristics of primers used in amplification reactions

Primer	Sequence 5′ → 3′	Position*	Annealing (°C)	Length (bp)
**Hamp-F**	GTCATTTATGGCCAAAAGTTTGCT	4409–4432	61	803
**Hamp-R**	CTCTCCCATCCCTGCTGC	5194–5211		

*Primers positions are assigned according to the NG_011563.2

### Statistical analysis

Statistical analysis was performed on IBM* SPSS* STATISTIC, 23.0 using independent t-test for the numeric variables and Fisher test for the nominal variables in which some categories had less than 5 cases. Any value less than 0.05 was considered statistically significant.

## Results

One hundred and two β-thalassemia major patients were recruited in this cohort study. Imaging and laboratory data of the patients has been shown in Table 2. According to TIF guideline, 71.3% of patients were categorized as iron overloaded [21]. The results of cardiac T2* classified the patients into normal (65.7%), mild (16.7%), moderate (10.8%), and severe (6.8%). For liver iron, these values were 11.7, 34.3, 21.6, and 6.9% respectively and 25.5% as very severe.

**Table 2:** Imaging and laboratory data of the patients based on the genotype of c.-582A > G variant

	AA/AG	GG
**Male Gender**	34.4%	50%
**Age**		
Mean ± SD	34.8 ± 6.1	31.5 ± 2.7
**TX (day)**		
Mean ± SD	18.4 ± 4.6	17.2 ± 3.0
**Hb (g/dl)**		
Mean ± SD	9.8 ± 1.2	8.7 ± 1.7
**Hct (%)**		
Mean ± SD	29.5 ± 3.2	29.3 ± 3.6
**Ferritin (ng/ml)**		
Median (25–75%)	1648.0 (773.8–3189.5)	2064.5 (1638.0–5518.0)
T2*MRI-H (ms)		
Mean ± SD	25.5 ± 9.1	17.3 ± 13.7
T2*MRI-L (ms)		
Mean ± SD	7.9 ± 9.3	2.2 ± 0.2

TX, transfusion intervals; Hb, hemoglobin; Hct, hematocrit; T2*MRI-H, heart iron concentration evaluated by MRI (normal rang is > 20 ms); T2*MRI-L, Liver iron concentration evaluated by MRI (normal range is > 6.3 ms)

We analyzed three SNPs of the HAMP gene located in the promoter region. Table 3 represents the genotyping results. Allele frequencies were calculated and were in agreement with Hardy-Weinberg equilibrium. The frequency of the minor allele (G) of c.-582A > G was determined to be 0.26. For c.-153C > T, all samples were homozygous for allele C. We also analyzed SNP c.-443C > T and found the frequency of the minor allele (T) to be 0.05.

**Table 3:** Genotyping result of c.-582A > G, c.-153C > T, and c.-443C > T variants

SNP	Genotype	No.	Percentage	Hardy-Weinberg equilibrium ***p*** value*
**c.-582A > G** (rs10421768)	AA	55	53.9	0.648
	AG	41	40.2	
	GG	6	5.9	
**c.-153C > T** (rs142126068)	CC	102	100	NA
	CT	0	0	
	TT	0	0	
**c.-443C > T** (rs117345431)	CC	92	90.2	0.602
	CT	10	9.8	
	TT	0	0	

*NA* not applicable

*Values more than 0.05 indicate that the population is in Hardy-Weinberg equilibrium

Our findings revealed a statistically significant difference between the level of cardiac iron accumulation and c.-582A > G variant (*p* = 0.02). Association between this variant and hepatic iron concentration was on the edge of significance (*p* = 0.051) while no association was found for ferritin level.

For c.-443C > T statistical analysis was on the edge of significant between the minor allele and serum ferritin (*p* = 0.058) but no correlation was found between this SNP and liver/cardiac iron accumulation (data not shown).

## Discussion

Hepcidin is an important regulator of iron homeostasis, which is involved in various metabolic pathways of iron metabolism [23]. It has been shown that certain polymorphisms in the HAMP promoter region may decrease the expression of this hormone and consequently increasing the serum iron [18, 19].

In this study, which is the first molecular analysis of hepcidin gene in Iran, we investigated the relationship between hepcidin promoter gene (HAMP) variants c.-582A > G, c.-153C > T, and c.-443C > T and iron overload in β-thalassemia major patients who had a regular transfusion and iron-chelating therapy.

Our study has shown that GG genotype of c.-582A > G variant is associated with a significant degree of cardiac iron overload. The CT genotype of c.-443C > T variant is also on the edge of significance with ferritin level. All patients were homozygotes for the normal allele (c.-153C), so no statistical analysis was possible.

Although all of the patients were under the standard protocol of iron-chelating therapy, most of them proved to be refractory which may be attributed to different above-mentioned genetic variations.

Parajes et al found that c.-582A > G variant is located in E-box 1 with a conserved sequence of CANNTG. This box is a responsive element for upstream stimulatory factors 1and 2 (USF1/USF2) and cMyc/Max heterodimers. When A is substituted by G, the transcription factors would not be able to bind the E-box sufficiently leading to decrease transcription of the HAMP gene [20]. This is compatible with our finding that homozygous patients for G allele had much more iron deposition in cardiac tissue (*p* = 0.02). Furthermore, serum ferritin level was evaluated and all patients with GG genotype had ferritin above 1000 ng/ml but no association was found between this SNP and serum ferritin level (*p* = 0.12). This may be explained by our small sample size.

The second variant we genotyped was c.-153C > T with the very low frequency of the minor allele in the population. It had primarily been identified by Island et al in a patient with massive iron overload. They demonstrated that this substitution, which is located in a BMP-responsive element, reduced basal hepcidin gene expression by impairing its response to BMPs and IL-6 [19]. In our study, we could not detect any T allele of this SNP in 102 patients. Different racial characteristics may explain the discrepancy between our results and others. On the other hand, Island et al found only one heterozygous person for this variant, so it may be just a mutation and not a polymorphism.

The third variant, c.-443C > T, was polymorphic just in 10 patients. Statistical analysis was on the edge of a significant relationship between c.-443C > T and serum ferritin (*p* = 0.058) but no association was found between this SNP and liver/cardiac iron concentration. All subjects heterozygous for this variant had at least one G allele of c.-582A > G variant. The proximity of these two SNPs may cause them to inherit as a haplotype. To the best of our knowledge, this SNP has not been evaluated in thalassemia by other investigators.

The results of this study can justify the fact that despite regular use of chelating drugs, even in high dose, cannot prevent iron accumulation in liver/cardiac in some patients. This can be attributed, at least in part, to the genetic background of patients. The use of mini-hepcidin may be an alternative to a high dose of chelating therapy in these patients.

## Conclusions

Iron metabolism is a complicated and fine-regulated process in health and disease. In thalassemia where there is an increased intake of iron through blood transfusion, this process is even more complicated. Our findings indicate that the outcome of iron-chelating therapy is strongly influenced by the genetic background of the patient and that polymorphisms in the hepcidin gene, especially those in the promoter region, play an important role in iron overload.

## Data Availability

Sequencing data of 102 blood samples aligned and analyzed on the platform of HAMP gene using NCBI accession number NG_011563.2 https://www.ncbi.nlm.nih.gov/nuccore/1543053630/

## Abbreviations

SNP: Single-Nucleotide Polymorphism
PCR: Polymerase Chain Reaction
IVS: Intervening Sequence
FPN: Ferroportin
ERFE: Erythroferrone
BMP: Bone Morphogenetic Protein
IL-6: Interleukin 6
TIF: Thalassemia International Federation
MRI: Magnetic Resonance Imaging
EDTA: Ethylenediaminetetraacetic Acid
WBC: White Blood Cell
USF: Upstream Stimulatory Factor

## Footnotes

1 Thalassemia International Federation
